# Additive interaction of mid- to late-life depression and cerebrovascular disease on the risk of dementia: a nationwide population-based cohort study

**DOI:** 10.1186/s13195-021-00800-z

**Published:** 2021-03-16

**Authors:** Yoo Jin Jang, Cinoo Kang, Woojae Myung, Shinn-Won Lim, Young Kyung Moon, Ho Kim, Doh Kwan Kim

**Affiliations:** 1 Department of Psychiatry, Samsung Medical Center, Sungkyunkwan University School of Medicine, 81 Irwon-ro, Gangnam-gu, Seoul, 06351 South Korea; 2 Department of Public Health Science, Graduate School of Public Health, Seoul National University, Seoul, South Korea; 3 Department of Neuropsychiatry, Seoul National University Bundang Hospital, Seongnam, South Korea; 4 SAIHST, Sungkyunkwan University School of Medicine, Seoul, South Korea; 5 Institute of Health and Environment, Seoul National University, 1 Gwanak-ro, Gwanak-gu, Seoul, 151-742 South Korea

**Keywords:** Dementia, Depression, Cerebrovascular disease, Alzheimer’s disease, Additive interaction, Risk factors, Nationwide population, Cohort study

## Abstract

**Background:**

Dementia is a progressive neurocognitive disease with a substantial social burden. No apparent breakthroughs in treatment options have emerged so far; thus, disease prevention is essential for at-risk populations. Depression and cerebrovascular disease (CVD) are independent risk factors for dementia, but no studies have examined their interaction effect on dementia risk. This study aimed to identify the association of depression and CVD with the risk of dementia and evaluate whether dementia risk among patients with comorbid depression and CVD is higher than the sum of the individual risk due to each condition.

**Methods:**

A population-based cohort study was conducted to analyze the Korean National Health Insurance Service-National Sample Cohort data of all individuals over 50 years of age. Individuals who had not been diagnosed with dementia at baseline were included and followed up from January 1, 2005, to December 31, 2013. A time-varying Cox proportional hazard regression model adjusted for potential confounding factors was used for the analysis. The interaction between depression and CVD was estimated based on the attributable proportion (AP), relative excess risk due to interaction (RERI), synergy index (SI), and multiplicative-scale interaction.

**Results:**

A total of 242,237 participants were included in the analytical sample, of which 12,735 (5.3%) developed dementia. Compared to that for participants without depression or CVD, the adjusted hazard ratio for the incidence of dementia for those with depression alone was 2.35 (95% confidence interval [CI] 2.21–2.49), CVD alone was 3.25 (95% CI 3.11–3.39), and comorbid depression and CVD was 5.02 (95% CI 4.66–5.42). The additive interaction between depression and CVD was statistically significant (AP—0.08, 95% CI 0.01–0.16; RERI—0.42, 95% CI 0.03–0.82; SI—1.12, 95% CI 1.01–1.24). The multiplicative interaction was significant too, but the effect was negative (0.66, 95% CI 0.60–0.73).

**Conclusions:**

In this population-based nationwide cohort with long-term follow-up, depression and CVD were associated with an increased risk of dementia, and their coexistence additively increased dementia risk more than the sum of the individual risks.

## Background

Dementia is a neurodegenerative disease characterized by progressive cognitive decline. It precludes patients from carrying out daily life activities independently and often has devastating effects on the lives of patients and their caregivers. Although the prevalence and disease burden of dementia are increasing [1], there have been no apparent breakthroughs in terms of treatment options to date. Therefore, identifying at-risk populations and providing tailored care are essential for cost-effective public health management [2].

Depression has been suggested as a risk factor for dementia. According to recent meta-analyses of several epidemiological studies [3, 4], late-life depression is associated with increased dementia risk. Some researchers have proposed indirect evidence that the effect of depression on dementia risk is altered by comorbid cerebrovascular disease (CVD), which is typically characterized by cerebral ischemia and hemorrhage. In a recent large-scale retrospective study that included 35,791 individuals with 13 years of follow-up, depression was found to have an exceptionally significant effect on dementia in individuals with incident stroke [5]. A systemic review has reported that cognitive decline in late-life depression is associated with vascular dysfunction, including white matter hyperintensity [6]. Reciprocally, risk factors for dementia in patients with CVD also include depressive illness [7]. Armstrong et al. conducted a longitudinal study and reported that late-life depression partially mediates the association between cardiovascular disease and cognitive decline [8].

However, the interaction effect of depression and CVD, that is, whether one disease strengthens the association of another with dementia risk, remains unclear. In contrast to dementia, the prevention and treatment of these two illnesses have been well established and are known to be effective [9, 10]. Therefore, clarifying the interaction effect between depression and CVD on dementia risk can help identify individuals who can benefit more from a health care service at the same cost.

In the current study, we sought to evaluate the association of depression and CVD with subsequent dementia using a nationwide population-based cohort. We also aimed to investigate whether the interaction between the two conditions is additively associated with increased dementia risk. As the epidemiology of depression, CVD, and dementia is dependent upon age and sex, we examined whether the interaction between depression and CVD differed by age or sex.

## Methods

### Study Population

We used the nationwide population-based South Korean National Health Insurance Service-National Sample Cohort (NHIS-NSC) database [11], which contains the data of approximately 1 million people (2.2% of the total Korean population) and uses systematic stratified random sampling with proportional allocation within each stratum (age, sex, insurance eligibility status, and income level). As the National Health Insurance (NHI) program requires mandatory health insurance for all Korean citizens, the database is representative of the South Korean population. All insurance claims are cataloged in this system, and the medical information in the database is provided exclusively by healthcare providers. Every NHI member older than 40 years is eligible for biennial cardiovascular health panel screening and cancer screening for free or at a minimal cost depending on income [12]. Prior studies have validated the NHIS-NSC data for some chronic diseases, including stroke and dementia [13–16]. Yet, the validation of the International Classification of Diseases, 10th revision (ICD-10) codes for depression diagnosis has not been evaluated [17].

**Fig. 1:**
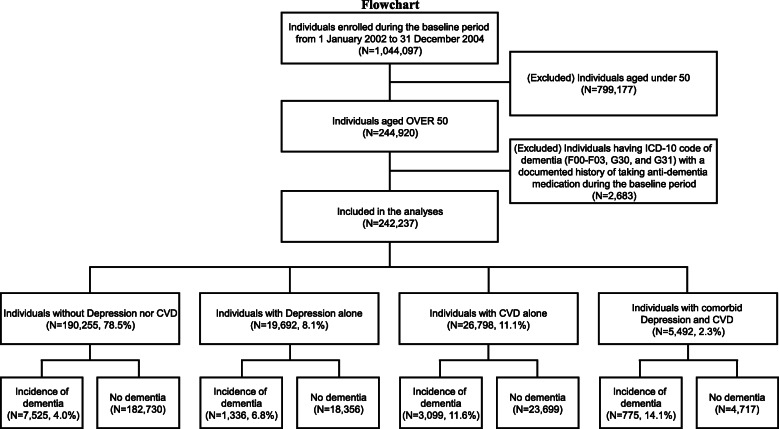
Study population flow diagram

Of the 1,044,097 individuals who were enrolled in the cohort during the baseline period (January 1, 2002, to December 31, 2004), we included 244,920 individuals over the age of 50 years and then excluded 2683 who had or received a dementia diagnosis during this period (Fig. 1). We followed up the participants from the index date (January 1, 2005) until December 31, 2013, or until the date of dementia onset within that period, defined as the follow-up period. Patient death and the end of the follow-up were treated as censoring events in the analyses. During the follow-up period of up to 9 years, individuals were classified into the following four groups based on their depression or CVD diagnosis: individuals with neither depression nor CVD, those with depression alone, those with CVD alone, and those with both depression and CVD.

The final analyses included a total of 242,237 participants. The institutional review board of the Samsung Medical Center, Seoul, South Korea, approved this study. All data were anonymized and kept confidential, and thus, the need for obtaining participant informed consent was waived.

### Exposure variables: depression and CVD

Depression was defined as the assignment of an ICD-10 code for depressive disorder (F32 or F33) and the documented administration of antidepressant medication (Additional file 1: Table S1) from the first day of depression diagnosis during the follow-up period as previously described [18–20]. CVD was defined as the assignment of an ICD-10 code for CVD (I60–69) as a primary diagnosis and two or more hospital visits during the follow-up period [20]. Unlike the medications used for depression or dementia, CVD medications are not exclusive to the disease. Therefore, we defined CVD based on the ICD codes and multiple hospital visits regardless of the medications prescribed.

### Outcome of interest: dementia

The primary outcome was overall survival free of dementia. We defined dementia as the assignment of an ICD-10 code for dementia (F00–03, G30–31) and the documented administration of anti-dementia medication (donepezil, rivastigmine, galantamine, or memantine) during the follow-up period [18, 21, 22]. As mentioned above, we excluded all cases with a dementia diagnosis during the baseline period to focus on incident cases of dementia. Dementia subtype was set as the secondary outcome and was defined based on the initial ICD-10 code assigned on the first day of dementia diagnosis: F00 and G30 for Alzheimer’s disease (AD), F01 for vascular dementia (VD), and F02-03 and G31 for other dementias (non-AD or non-VD).

### Covariates

All analyses were adjusted for potential confounding variables—age, sex, residential area, income level, and comorbidities—during the baseline period from 2002 to 2004. With regard to the residential area, the capital city of South Korea and the surrounding metropolitan cities (Seoul, Incheon, Gyeonggi-do) were designated as the “capital region”; all other regions were designated as “non-capital regions.” Income levels were classified as low (up to the 30th percentile), middle (30th to 70th percentile), and high (70th to 100th percentile). The ICD-10 codes from the Charlson comorbidity index, which is used widely to adjust for the effects of comorbidities [23], were used. We defined the comorbidities based on the ICD-10 codes for each disease (Additional file 1: Table S2) and two or more hospital visits during the baseline period.

### Interaction measurements

We assessed additive- and multiplicative-scale interaction measures to examine the interaction effect of depression and CVD on dementia onset. In terms of the additive interaction, we derived the attributable proportion due to interaction [AP; Eq. (1)], relative excess risk due to interaction [RERI; Eq. (2)], and synergy index [SI; Eq. (3)]. The AP is the proportion of the risk due to the interaction in the doubly exposed group (null hypothesis: AP = 0). When RERI is positive, it indicates increased risk due to the additive interaction (null hypothesis: RERI = 0). SI can be interpreted as the ratio of an increased risk due to both exposures to the sum of individual increased risks (null hypothesis: SI = 1). These were used to assess whether the risk due to having both diseases is greater than the sum of the risks due to each condition [24, 25].

1 $$ {\mathrm{AP}}_{\mathrm{Interaction}}=\frac{{\mathrm{HR}}_{\mathrm{Depression}\&\mathrm{CVD}}-{\mathrm{HR}}_{\mathrm{Depression}}-{\mathrm{HR}}_{\mathrm{CVD}}+1}{{\mathrm{HR}}_{\mathrm{Depression}\&\mathrm{CVD}}} $$

2 $$ {\mathrm{RERI}}_{\mathrm{Interaction}}={\mathrm{HR}}_{\mathrm{Depression}\&\mathrm{CVD}}-{\mathrm{HR}}_{\mathrm{Depression}}-{\mathrm{HR}}_{\mathrm{CVD}}+1 $$

3 $$ {\mathrm{SI}}_{\mathrm{Interaction}}=\frac{{\mathrm{HR}}_{\mathrm{Depression}\&\mathrm{CVD}}-1}{{\mathrm{HR}}_{\mathrm{Depression}}+{\mathrm{HR}}_{\mathrm{CVD}}-2} $$

The multiplicative-scale interaction [Eq. (4)] has been widely used to examine the interaction effects by identifying whether the risk due to having both diseases is greater than the product of the risks due to each disease alone (null hypothesis: multiplicative interaction = 1) [24, 25].

4 $$ {\mathrm{Mult}}_{\mathrm{Interaction}}=\frac{{\mathrm{HR}}_{\mathrm{Depression}\&\mathrm{CVD}}}{{\mathrm{HR}}_{\mathrm{Depression}}\times {\mathrm{HR}}_{\mathrm{CVD}}} $$

In Eqs. (1–4), HR_Depression & CVD_ represents the hazard ratio (HR) for those who have both depression and CVD. HR_Depression_ and HR_CVD_ represent the HR for those who have depression or CVD alone, respectively, compared with individuals who have neither of the conditions.

### Statistical analysis

We used Cox proportional hazards regression models to determine the adjusted hazard ratios (aHRs) and 95% confidence intervals (CIs) of depression, CVD, or comorbid depression and CVD for dementia incidence. As the cohort design can cause an immortal time bias, we used a time-varying Cox regression model to prevent time-related biases [26, 27]. An unadjusted time-varying Cox regression analysis was performed (model 1), followed by a demographic characteristics-adjusted (model 2; adjusted for age, sex, residential area, and income level), and a comorbidity-adjusted (model 3; adjusted for myocardial infarction, congestive heart failure, peripheral vascular disease, chronic pulmonary disease, connective tissue disorder, peptic ulcer, mild liver disease, uncomplicated diabetes, complicated diabetes, hemiplegia, moderate or severe renal diseases, non-metastatic solid cancer, moderate or severe liver diseases, and metastatic solid cancer in addition to the demographic characteristics in model 2) analysis. The proportional hazards assumption was graphically tested and verified using the Schoenfeld residual method; no variables violated the assumption.

First, we used a log-rank test and evaluated independent associations of depression and CVD with subsequent dementia using aHRs and 95% CIs in two separate regression models. In this analysis, exposure of interest (depression or CVD) was treated as a time-varying variable, and the other comorbid illnesses were regarded as time-fixed confounders to be adjusted. Next, we examined the interaction effect of the two exposure diseases by calculating the additive (AP, RERI, and SI) and multiplicative interaction. We verified the significance of the interaction term and then stratified each subgroup based on age or sex. The two-way interaction effect was tested on independent associations of depression or CVD with each subgroup, and the three-way interaction effect was tested on interactive associations of depression and CVD with each subgroup. We then conducted a subgroup analysis using a fully adjusted Cox regression model (model 3; demographic characteristics and comorbidities adjusted) in which dementia subtypes (AD, VD, and non-AD or non-VD) were accounted into the outcome variables.

We also carried out sensitivity analyses to ensure the robustness of the results. First, a lagged-time analysis was conducted because depression that occurs shortly before dementia onset can be a prodrome of dementia [28]. We classified individuals who were newly diagnosed with depression during the lagged-time period into the “no depression” group. Second, we repeated the analysis using ICD-10 code disease definitions only. As the strict operational definitions of depression, CVD, and dementia can lead to selection bias, we applied mitigated definitions regardless of the medication prescriptions or the number of hospital visits. All statistical analyses were performed using SAS 9.4 (SAS Institute Inc., Cary, NC, USA).

## Results

### Participant characteristics and incidence of dementia

Participant characteristics are presented in Table 1. A total of 242,237 participants were included in the analyses (77,587 [68.0%] aged over 65 years at baseline; 131,712 [54.4%] women; 140,415 [58.0%] from non-capital regions). A total of 7006 (2.9%) participants had histories of depression during the baseline period, and 9680 (4.0%) participants had a history of CVD during the same period. The demographic characteristics of the study participants and the diseases from the Charlson comorbidity index were set as covariates (Table 1). During the follow-up period, 12,735 (5.3%) participants were newly diagnosed with dementia (AD, 9729 [76.4%]; VD, 1306 [10.3%]; non-AD or non-VD, 1700 [13.3%]; Additional file 1: Table S3). There were significant differences between the groups in terms of age, sex, income level, and comorbidities (*p* value < 0.05).

**Table 1:** Descriptive characteristics of the study population

	Study population		With dementia		Without dementia	
	*N*	%	*N*	%	*N*	%
**Total**	242,237	100.0	12,735	100.0	229,502	100.0
**Age**						
50 to 64 years	164,650	68.0	3345	26.3	161,305	70.3
Above 64 years	77,587	32.0	9390	73.7	68,197	29.7
**Sex**						
Men	110,525	45.6	3982	31.3	106,543	46.4
Women	131,712	54.4	8753	68.7	122,959	53.6
**Residential area**^**a**^						
Capital region	101,822	42.0	4403	34.6	97,419	42.5
Non-capital region	140,415	58.0	8332	65.4	132,083	57.6
**Income level**^**b**^						
Low	65,730	27.1	3948	31.0	61,782	26.9
Middle	80,753	33.3	3683	28.9	77,070	33.6
High	95,754	39.5	5104	40.1	90,650	39.5
**Comorbidities**						
Myocardial infarction	2332	1.0	156	1.2	2176	1.0
Congestive heart failure	8904	3.7	814	6.4	8090	3.5
Peripheral vascular disease	6661	2.8	575	4.5	6086	2.7
Chronic pulmonary disease	47,871	19.8	3232	25.4	44,639	19.5
Connective tissue disorder	11,761	4.9	881	6.9	10,880	4.7
Peptic ulcer	50,849	21.0	2984	23.4	47,865	20.9
Mild liver disease	22,914	9.5	1022	8.0	21,892	9.5
Uncomplicated diabetes	29,720	12.3	2209	17.4	27,511	12.0
Complicated diabetes^c^	11,717	4.8	933	7.3	10,784	4.7
Hemiplegia	1533	0.6	151	1.2	1382	0.6
Moderate or severe renal diseases	1476	0.6	96	0.8	1380	0.6
Non-metastatic solid cancer^d^	9382	3.9	435	3.4	8947	3.9
Moderate or severe liver diseases	754	0.3	17	0.1	737	0.3
Metastatic solid cancer	2805	1.2	51	0.4	2754	1.2
Depression	7006	2.9	652	5.1	6354	2.8
Cerebrovascular disease	9680	4.0	1007	7.91	8673	3.8

^a^Individuals who resided in the capital city of South Korea and surrounding metropolitan cities (Seoul, Incheon, Gyeonggi-do) were classified as belonging to the “capital region” and the others to the “non-capital region”

^b^Income levels were divided into three groups: low (up to the 30th percentile), middle (30th to 70th percentile), and high (70th to 100th percentile) income

^c^Diabetes complicated with retinopathy, neuropathy, or renal disease

^d^Non-metastatic solid cancer, including leukemia, lymphoma, and multiple myeloma

When the patients were classified into the four categories as shown in Fig. 1, the number of patients with newly diagnosed dementia was 7525 (4.0%) in the reference group (*N* = 190,255; 78.5%), 1336 (6.8%) in the depression alone group (*N* = 19,692; 8.1%), 3099 (11.6%) in the CVD alone group (*N* = 26,798; 11.1%), and 775 (14.1%) in the comorbid depression and CVD group (*N* = 5492; 2.3%).

### Independent associations of depression and CVD with increased dementia risk

**Fig. 2:**
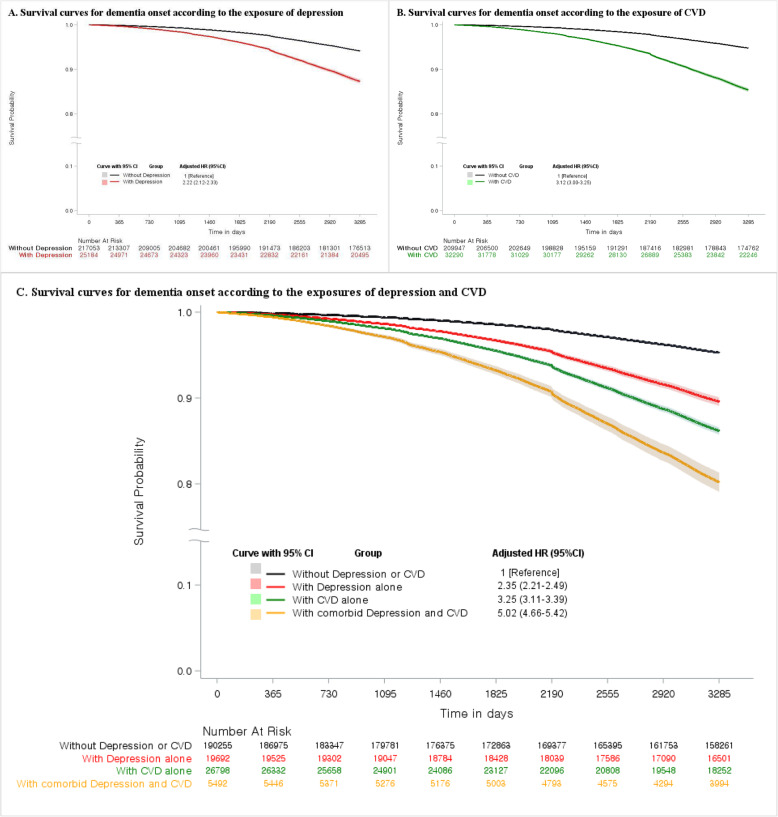
Survival curves for dementia onset according to exposure diseases. **a** Survival curves for dementia onset according to the exposure of depression. **b** Survival curves for dementia onset according to the exposure of CVD. **c** Survival curves for dementia onset according to the exposures of depression and CVD

We found significant associations of depression and CVD with the risk of dementia (Additional file 1: Figure S1). In the time-varying Cox proportional hazard model analysis, depression was associated with a 122% increased risk of dementia (aHR 2.22, 95% CI 2.12–2.33; Table 2, Fig. 2a) after adjusting for age, sex, residential area, income level, and comorbid chronic diseases, compared to participants without depression. The lagged-time analyses showed that the effect of depression was significant even after considering depression that occurred within the lagged-time period up to 2 years as a prodrome of dementia (Additional file 1: Table S4). The analyses with CVD showed that it was associated with a higher hazard for dementia onset (aHR 3.12, 95% CI 3.00–3.25; Table 2, Fig. 2b). Significant effect modifications were observed according to age and sex subgroups in both the analyses for depression and CVD patients (all *p* values for the two-way interactions between diseases and age or sex were <0.0001). The association between depression and subsequent dementia was especially high in those under the age of 65 and men, and the same was observed for CVD (Table 2).

**Table 2:** Cox regression analysis for independent associations between depression/CVD and dementia

	No depression	Depression	No CVD	CVD
**Total population**	217,053 (89.6%)	25,184 (10.4%)	209,947 (86.7%)	32,290 (13.3%)
Dementia events	10,624 (4.9%)	2111 (8.4%)	8861 (4.2%)	3874 (12.0%)
Person-years	1,777,860	210,499	1,734,836	253,523
Incidence (events/1000 person-years)	5.98	10.03	5.11	15.28
Log-rank test (*p* value)	< 0.0001		< 0.0001	
Unadjusted HR in model 1 (95% CI)	1 [reference]	2.40 (2.29–2.51)	1 [reference]	4.36 (4.20–4.53)
aHR in model 2 (95% CI) ^a^	1 [reference]	2.35 (2.24–2.46)	1 [reference]	3.26 (3.14–3.39)
aHR in model 3 (95% CI) ^b^	1 [reference]	2.22 (2.12–2.33)	1 [reference]	3.12 (3.00–3.25)
**Age 50 to 64 years**	147,019 (89.3%)	17,631 (10.7%)	147,069 (89.3%)	17,851 (10.7%)
Dementia events	2591 (1.8%)	754 (4.3%)	2150 (1.5%)	1195 (6.8%)
Person-years	1,271,789	152,534	1,276,238	148,084
Incidence (events/1000 person-years)	2.04	4.94	1.68	8.07
Log-rank test (*p* value)	< 0.0001		< 0.0001	
Unadjusted HR in model 1 (95% CI)	1 [reference]	3.43 (3.16–3.72)	1 [reference]	6.82 (6.35–7.32)
aHR in model 2 (95% CI)^a^	1 [reference]	3.24 (2.99–3.52)	1 [reference]	6.84 (6.37–7.34)
aHR in model 3 (95% CI)^b^	1 [reference]	3.11 (2.86–3.37)	1 [reference]	6.54 (6.09–7.03)
**Age above 64 years**	70,034 (90.3%)	7553 (9.7%)	62,878 (81.0%)	14,709 (19.0%)
Dementia events	8033 (11.5%)	1357 (18.0%)	6711 (10.7%)	2679 (18.2%)
Person-years	506,071	57,965	458,597	105,439
Incidence (events/1000 person-years)	15.87	23.41	14.63	25.41
Log-rank test (*p* value)	< 0.0001		< 0.0001	
Unadjusted HR in model 1 (95% CI)	1 [reference]	2.06 (1.95–2.18)	1 [reference]	2.50 (2.39–2.62)
aHR in model 2 (95% CI)^a^	1 [reference]	2.04 (1.93–2.16)	1 [reference]	2.55 (2.43–2.66)
aHR in model 3 (95% CI)^b^	1 [reference]	1.92 (1.81–2.04)	1 [reference]	2.44 (2.33–2.56)
**Men**	102,043 (92.3%)	8482 (7.7%)	95,637 (86.5%)	14,888 (13.5%)
Dementia events	3388 (3.3%)	594 (7.0%)	2531 (2.7%)	1451 (9.6%)
Person-years	831,452	69,492	784,992	115,952
Incidence (events/1000 person-years)	4.07	8.55	3.22	12.51
Log-rank test (*p* value)	< 0.0001		< 0.0001	
Unadjusted HR in model 1 (95% CI)	1 [reference]	3.13 (2.87–3.42)	1 [reference]	5.69 (5.33–6.07)
aHR in model 2 (95% CI)^a^	1 [reference]	2.83 (2.60–3.09)	1 [reference]	4.15 (3.88–4.42)
aHR in model 3 (95% CI)^b^	1 [reference]	2.69 (2.46–2.93)	1 [reference]	3.99 (3.74–4.26)
**Women**	115,010 (87.3%)	16,702 (12.7%)	114,310 (86.8%)	17,402 (13.2%)
Dementia events	7236 (6.3%)	1517 (9.1%)	6330 (5.5%)	2423 (13.9%)
Person-years	946,408	141,007	949,844	137,571
Incidence (events/1000 person-years)	7.65	10.76	6.66	17.61
Log-rank test (*p* value)	< 0.0001		< 0.0001	
Unadjusted HR in model 1 (95% CI)	1 [reference]	1.97 (1.87–2.09)	1 [reference]	3.84 (3.67–4.03)
aHR in model 2 (95% CI)^a^	1 [reference]	2.19 (2.07–2.32)	1 [reference]	2.89 (2.76–3.03)
aHR in model 3 (95% CI)^b^	1 [reference]	2.07 (1.96–2.19)	1 [reference]	2.77 (2.64–2.91)

*Abbreviations*: *aHR* adjusted hazard ratio, *CI* confidence interval, *CVD* cerebrovascular disease, *HR* hazard ratio

^a^Adjusted for demographic characteristics (age, sex, residential area, and income level)

^b^Adjusted for demographic characteristics (age, sex, residential area, and income level), other exposure diseases, and 14 comorbidities (myocardial infarction, congestive heart failure, peripheral vascular disease, chronic pulmonary disease, connective tissue disorder, peptic ulcer, mild liver disease, uncomplicated diabetes, complicated diabetes, hemiplegia, moderate or severe renal diseases, non-metastatic solid cancer, moderate or severe liver diseases, and metastatic solid cancer)

### Interaction effect of depression and CVD on dementia risk

Compared to the no depression or CVD group, the depression alone group (aHR 2.35, 95% CI 2.21–2.49), CVD alone group (aHR 3.25, 95% CI 3.11–3.39), and comorbid depression and CVD group (aHR 5.02, 95% CI 4.66–5.42) were significantly associated with an increased risk of dementia (Table 3). All indices of the additive interaction were statistically significant with positive values (AP 0.08, 95% CI 0.01–0.16; RERI 0.42, 95% CI 0.03–0.82; SI 1.12, 95% CI 1.01–1.24; Table 3 and Fig. 2c). When participants were classified according to their dementia subtypes, significant additive interaction between depression and CVD was found with AD (AP 0.09, 95% CI 0.00–0.18; RERI 0.41, 95% CI − 0.01–0.83; SI 1.13, 95% CI 1.00–1.28; Table 3). VD or other dementia were not associated with the additive interaction effect of the two diseases. The effect modification by age was statistically insignificant. However, the interaction effect between depression and CVD changed with sex and was significant (*p* value for the three-way interaction among diseases and sex was < 0.0001) only in women (AP 0.12, 95% CI 0.03–0.21; RERI 0.53, 95% CI 0.11–0.95; SI 1.18, 95% CI 1.04–1.34; Table 3). The multiplicative interaction was significant in every analysis, but the effect was negative (Table 3). The sensitivity analysis using disease definitions based on the ICD-10 codes further supported the results (Additional file 1: Table S5).

**Table 3:** Interaction effect of depression and CVD on the risk of dementia onset

	Risk of dementia by exposure, HR (95% CI)				Additive interaction (95% CI)			Multiplicative interaction^a^ (95% CI)
	No depression or CVD	Depression	CVD	Depression and CVD	AP^a^	*RERI*^a^	SI^a^	
**Model 1**^**b**^	1 [Reference]	2.41 (2.27–2.55)	4.51 (4.32–4.70)	6.54 (6.08–7.05)	0.10 (0.02–0.17)	0.63 (0.13–1.13)	1.13 (1.03–1.24)	0.60 (0.55–0.67)
**Model 2**^**c**^	1 [Reference]	2.39 (2.26–2.54)	3.33 (3.19–3.47)	5.16 (4.79–5.56)	0.09 (0.01–0.16)	0.44 (0.04–0.84)	1.12 (1.01–1.24)	0.65 (0.59–0.71)
**Model 3**^**d**^	1 [Reference]	2.35 (2.21–2.49)	3.25 (3.11–3.39)	5.02 (4.66–5.42)	0.08 (0.01–0.16)	0.42 (0.03–0.82)	1.12 (1.01–1.24)	0.66 (0.60–0.73)
**Dementia subtype**^**e**^								
AD	1 [Reference]	2.36 (2.21–2.52)	2.77 (2.63–2.91)	4.54 (4.15–4.95)	0.09 (0.00–0.18)	0.41 (−0.01–0.83)	1.13 (1.00–1.28)	0.69 (0.62–0.78)
VD	1 [Reference]	2.13 (1.68–2.70)	9.75 (8.64–11.02)	11.98 (9.74–14.75)	0.09 (− 0.14–0.32)	1.1 (−1.29–3.49)	1.11 (0.89–1.39)	0.58 (0.42–0.78)
Non-AD or non-VD^f^	1 [Reference]	2.37 (2.02–2.78)	2.97 (2.64–3.35)	4.55 (3.68–5.62)	0.05 (− 0.18–0.28)	0.21 (− 0.80–1.22)	1.06 (0.80–1.42)	0.65 (0.49–0.85)
**Age**^**g**^								
50 to 64 years	1 [Reference]	3.46 (3.12–3.83)	6.93 (6.38–7.53)	10.10 (8.85–11.53)	0.07 (− 0.07–0.21)	0.72 (− 0.61–2.05)	1.09 (0.94–1.26)	0.42 (0.36–0.50)
Above 64 years	1 [Reference]	2.01 (1.87–2.16)	2.53 (2.41–2.66)	3.83 (3.50–4.21)	0.08 (− 0.02–0.18)	0.29 (− 0.08–0.67)	1.12 (0.97–1.28)	0.75 (0.67–0.85)
**Sex**^**g**^								
Men	1 [Reference]	3.16 (2.82–3.54)	4.31 (4.01–4.64)	6.71 (5.85–7.69)	0.04 (− 0.12–0.19)	0.24 (− 0.71–1.19)	1.04 (0.88–1.23)	0.75 (0.67–0.85)
Women	1 [Reference]	2.11 (1.97–2.26)	2.82 (2.67–2.97)	4.46 (4.07–4.88)	0.12 (0.03–0.21)	0.53 (0.11–0.95)	1.18 (1.04–1.34)	0.69 (0.62–0.78)

*Abbreviations*: *AD* Alzheimer’s disease, *AP* attributable proportion due to interaction, *CI* confidence interval, *CVD* cerebrovascular disease, *HR* hazard ratio, *RERI* relative excess risk due to interaction, *SI* synergy index, *VD* vascular dementia

^a^Null hypothesis for each interaction is AP = 0, RERI = 0, SI = 1, and multiplicative interaction = 1

^b^Unadjusted model

^c^Adjusted for demographic characteristics (age, sex, residential area, and income level)

^d^Adjusted for demographic characteristics (age, sex, residential area, and income level) and 14 comorbidities (myocardial infarction, congestive heart failure, peripheral vascular disease, chronic pulmonary disease, connective tissue disorder, peptic ulcer, mild liver disease, uncomplicated diabetes, complicated diabetes, hemiplegia, moderate or severe renal diseases, non-metastatic solid cancer, moderate or severe liver diseases, and metastatic solid cancer)

^e^Subgroup analysis for dementia subtype was based on model 3 (adjusted for demographic characteristics and 14 comorbidities) and conducted separately by considering the other dementia subtypes as a competing risk

^f^Includes dementia in other diseases classified elsewhere (F02), unspecified dementia (F03), other degenerative diseases of the nervous system, and not elsewhere classified (G31)

^g^Subgroup analyses for age and sex were based on model 3 (adjusted for demographic characteristics and 14 comorbidities). Age or sex was not considered as a covariate in each subgroup analysis

## Discussion

In this analysis of a nationwide population-based cohort of 242,237 participants aged over 50 years, depression and CVD were independently associated with a more than double the risk of dementia. After adjusting for demographic factors and comorbidities, we found a positive additive interaction between depression and CVD on dementia risk, and this interaction effect was significant only in women. In the analyses with dementia subtypes, the interaction effect was associated with AD.

Our results reinforce the claim that individuals with depression or CVD are more vulnerable to subsequent dementia. We replicated prior findings with a large population-based sample and also tested the generalizability of our results. In this study, the effect of depression on incident dementia was found to be stronger in individuals below the age of 65 years and men than that in individuals above the age of 64 years and women, respectively. Our finding that participants with depression that appeared in middle age are more vulnerable to subsequent dementia contradicts those of some studies [29, 30] that have reported that late-life depression is more strongly associated with dementia than mid-life depression. However, a few researchers have argued that early-onset (before the age of 65 years) depression may be a long-term risk factor for dementia [31]. Whether the effect of depression on dementia risk differs according to the age of onset or the duration of morbidity is controversial and needs further investigation. The effect modifications of sex on depression as a risk factor for AD reported by previous studies have also been inconsistent [32]. According to a recent systematic review [33], these discrepant findings may be accounted for by methodological differences, such as those related to the recruitment method (clinic-based or population-based), follow-up duration, and measure of depression used. The current results provide evidence that depression among men in a community-based population is more strongly associated with increased dementia risk than among women.

Individuals with CVD were more likely to be diagnosed with dementia, especially VD, with more than three times the risk, which is in line with the previous literature [34]. Moreover, CVDs in men and younger age groups were more strongly associated with increased dementia risk. This is consistent with previous studies which reported that post-stroke dementia is more prevalent in men [35]. In large-scale population-based cohort studies on the association of cardiovascular risk [36] and blood pressure pattern [37] with cognitive function, researchers have consistently reported that mid-life vascular disease has a more substantial effect on dementia onset. Our findings highlight the importance of midlife as a critical period for later cognitive function.

We found that both additive and multiplicative interactions of depression and CVD on dementia risk were statistically significant. However, they showed the opposite effect. This phenomenon is understood as the concept of “interaction continuum” [38]. The form of interaction depends on the relative magnitude of the probability of the outcome, since it is calculated based on each baseline subgroup risk; therefore, the scale of the risk (i.e., scale dependence) affects it. In our result, on the risk difference scale, the effect of depression was larger in the CVD group than in the non-CVD group (risk differences 1.77 vs. 1.35). Moreover, the effect of the CVD was larger for those who had depression than for the non-depression group (risk differences 2.67 vs. 2.25). However, on the risk ratio scale, the effect of depression was smaller in the CVD group than in the non-CVD group (risk ratios 1.77 vs. 2.35). This is because the baseline risks for the depression and CVD groups were relatively high, making it unlikely that the observed risk in the comorbid depression and CVD group would exceed the multiplication of these baseline risks (i.e., 2.35 × 3.25 = 7.64). Nevertheless, the existence of a positive additive interaction in the absence of multiplicative interaction is still meaningful [38]. VanderWeele and Knol [39] also reported that additive interaction, rather than multiplicative interaction, is more suitable and important to be assessed as a relevant public health measure because multiplicative-scale interaction without scale-dependent consideration sometimes indicates the wrong subgroup (in our case, “no depression nor CVD” group, but not “depression” or “CVD” group) for intervention or treatment. In this study, the negative multiplicative interaction effect may also be partially explained by the operational definition of medication use or multiple hospital visits (theoretically possible therapeutic or protective effect from the simultaneous presence of two diseases). Therefore, in terms of biological plausibility and from the public health perspective, a positive additive interaction seems more reasonable than a negative multiplicative interaction in the context of our study [40].

The positive additive interaction between depression and CVD on dementia risk remained statistically significant after adjusting for covariates. Although we cannot clarify the underlying biological mechanisms based on this study, one possible explanation is that each illness exacerbates the other condition through biological and psychosocial changes. We could not tell the severity of each disease in our analyses, but generally, patients accompanied by another disease are likely to be in a more severe condition and have poor outcomes. Stroke limits patients’ activities of daily living and impairs their social and cognitive functions. It frequently gives rise to a depressed mood as a psychosocial reaction [41]. Vascular damage in specific brain regions related to mood regulation could make patients susceptible to depressive disorders [42]. Besides, depression might affect patient health behaviors and make them vulnerable to CVD through HPA axis dysregulation or a chronic inflammatory state [43]. Another explanation for the additive interaction between depression and CVD is related to AD pathology. In the analysis of dementia subtypes, the interaction between depression and CVD was only associated with AD. Although it requires cautious interpretation, we hypothesize that the concurrence of vascular dysfunction from CVD and AD pathology triggered by depression initiates and aggravates clinically significant cognitive decline. In a recent clinical study on CVD and AD pathology with 218 participants, CVD was not associated with the rate of the beta-amyloid accumulation, but the comorbidity of CVD and amyloid plaques accumulation was associated with cognitive decline [44]. Chronic stress and hypercortisolemia can induce beta-amyloid accumulation, hyperphosphorylation of Tau proteins, and neurotoxicity [45]. A prior study reported that depressed patients have higher beta-amyloid aggregation levels than healthy individuals [46]. The additive interaction of depression and CVD on the dementia risk in our study was more remarkable in women, who are known to be at risk of AD [1]. Considering the age criteria (over 50 years) and common menopausal age in our study, the sex effect seems to be explained not only by the role of estrogen or other sex steroids alone, but also by comprehensive sex differences in brain aging [47]. A previous study that demonstrated the sex difference in risk factors for the transition from mild cognitive impairment to AD reported that severe periventricular white matter hyperintensities and poorer global cognitive function are the risk factors for men; however, depressive symptoms and genetic burden of apolipoprotein ε4 allele are the risk factors for women [48]. This result implies that the correlation between depression and dementia is different between men and women and is in line with accumulating evidence for sex differences in brain aging in our study. Further research is required to discover the underlying neurobiology of sex differences with respect to the effect of depression and CVD on dementia.

Our findings indicate that individuals with depression or CVD require thorough preventive approaches to avoid another illness. Although treating depressive disorder might not change the course of a patient’s cognitive decline [30], managing vascular risk factors and preventing CVD in individuals with depression could reduce the incidence of dementia and personal and socioeconomic disease burdens. Women are known to have a higher risk of depression; therefore, targeting this at-risk population can also help the dementia prevention policies to work more effectively.

### Limitations

This study has some limitations. The NHIS-NSC database contains health insurance claims data and not diagnostic data [20]. Therefore, it is possible that the disease diagnosis data used in this research were inaccurate, which may have resulted in misclassification of patients who did not seek treatment as healthy controls. We set up strict operational definitions using multiple hospital visits or drug prescription data in addition to the ICD-10 codes to validate the diagnoses. However, these criteria also cause a selection bias, so we would have accidentally excluded “real” patients who did not visit hospitals or missed their diagnosis owning to minimal symptoms. Second, the characteristics of the cohort could have influenced the results. The analyses were based on individuals who benefited from the universal healthcare system of South Korea; hence, our results have limited generalizability for other ethnicities and healthcare systems. Further, since the database did not include non-insurance benefits data [11], we missed out depressed patients who underwent non-pharmacological treatments, such as cognitive-behavioral therapy. Individuals who are on medications for depression or cognitive decline without being diagnosed can be considered rare because it is not recommended to prescribe medications without a relevant ICD-10 diagnosis in South Korea. Moreover, the prevalence of each disease in our sample was similar to that from other Korean registries [49–51], and the HRs for depression [3, 30, 52, 53] and CVD [54–57] were also similar to those previously reported. Third, the identification of dementia etiologies without biopsies or autopsies is complicated; therefore, we had no choice but to determine the dementia subtypes according to the initial codes of the main diagnoses for our analyses. Unfortunately, this method cannot differentiate between clinically noteworthy dementia etiologies that are not specified in a single ICD-10 code, such as “mixed dementia.” Another limitation is that possible confounding factors could be considered insufficient. Health-related behaviors, such as drinking and smoking, are known to affect depression, CVD, and dementia. They depend on age and sex; therefore, our findings with regard to effect modification by age and sex should be interpreted with caution. Finally, the selection of patients with CVD may have led to survival bias, which should be taken into account. Although CVD mortality has significantly decreased over the last 3 decades to 78% and 68% in men and women, respectively, in South Korea [58], it is still one of the top causes of mortality. However, CVD remains a great health burden for each patient and society, and chronic treatment including medication to prevent additional CVD for lifelong and rehabilitation to recover the function is needed. Hence, CVD can be regarded as a chronic disease, and survivor-related research is important as well.

## Conclusions

This nationwide, population-based cohort study demonstrates that both depression and CVD, as well as their interaction, are associated with an increased dementia risk. To the best of our knowledge, this is the first study to examine the coexistence effect of depression and CVD on subsequent dementia risk, and we found positive additive interaction between depression and CVD on the increased risk of dementia. Our results suggest that individuals with depression or CVD need interventions to prevent other conditions and that further investigation on the relevance of AD is required.

## Supplementary Information

**Additional file 1: Table S1.** Medications prescribed according to the respective dementia and depression ICD-10 codes. **Table S2.** ICD-10 code definitions of dementia, depression, CVD, and 14 other diseases from the Charlson comorbidity index. **Table S3.** Observed results according to dementia subtype. **Table S4.** Lagged-time analysis result considering depression as a misdiagnosis or a prodrome of dementia. **Table S5.** Sensitivity analysis for the interaction effect of depression and CVD on the risk of dementia onset when depression, CVD, and dementia were defined only by the ICD-10 codes (not considering medication use). **Figure S1.** Unadjusted survival curves for dementia onset according to exposure diseases.

## Data Availability

The data that support the findings of this study are available from the Korean National Health Insurance Service (NHIS), but restrictions apply to the availability of these data, which were used under license for the current study, and so are not publicly available. Data are however available from the authors upon reasonable request and with permission from the NHIS.

## Abbreviations

AD: Alzheimer’s disease
aHR: Adjusted hazard ratio
AP: Attributable proportion
CI: Confidence interval
CVD: Cerebrovascular disease
HPA: Hypothalamic-pituitary-adrenal
HR: Hazard ratio
ICD-10: International Classification of Diseases, 10th revision
NHI: National Health Insurance
NHIS-NSC: National Health Insurance Service-National Sample Cohort
RERI: Relative excess risk due to interaction
SI: Synergy index
VD: Vascular dementia
