# Supplementary material for: Additive interaction of mid- to late-life depression and cerebrovascular disease on the risk of dementia: a nationwide population-based cohort study
Source: Alzheimers Res Ther. 2021 Mar 16;13:61. doi: 10.1186/s13195-021-00800-z (PMC7968260; doi:10.1186/s13195-021-00800-z)

**Additive interaction of mid- to late-life depression and cerebrovascular disease on the risk of dementia: A nationwide population-based cohort study**

Yoo Jin Jang^1†^, Cinoo Kang^2†^, Woojae Myung^3^, Shinn-Won Lim^4^, Young Kyung Moon^1^, Ho Kim^5*^, and Doh Kwan Kim^1*^

**1. Supplementary tables**

**Table S1** Medications prescribed according to the respective dementia and depression ICD-10 codes................2

**Table S2** ICD-10 code definitions of dementia, depression, CVD, and 14 other diseases from the Charlson comorbidity index....................................................................................................................................................3

**Table S3** Observed results according to dementia subtype……............................................................................4

**Table S4** Lagged-time analysis result considering depression as a misdiagnosis or a prodrome of dementia......4

**Table S5** Sensitivity analysis for the interaction effect of depression and CVD on the risk of the dementia onset when depression, CVD, and dementia were defined only by the ICD-10 codes (not considering medication use) with at least 1 hospital visit...........................................................................................................................................................................5

**2. Supplementary figure**

Fig. S1 Unadjusted survival curves for dementia onset according to exposure diseases……....................................6

**Table S1** Medications prescribed according to the respective dementia and depression ICD-10 codes

| **Condition** | **Name** | **Drug Code** |
| --- | --- | --- |
| **Outcome disease** | |  |
| **Dementia** | donepezil | 148601ATB 148601ATD 148602ATB 148602ATD 148603ATB |
|  | rivastigmine | 224501ACH 224502ALQ 224503ACH 224504ACH 224505ACH 224506CPC 224507CPC 224508CPC |
|  | galantamine | 385201ATB 385202ATB 385203ACR 385203ATR 385204ACR 385204ATR 385205ACR 385205ATR |
|  | memantine | 190001ALQ 190001ATB 190001BIJ 190002ASY |
| **Exposure Disease** | |  |
| **Depression** | escitalopram | 474801ATB 474802ATB 474803ATB 474804ATB 521101ATD 521102ATD |
|  | fluoxetine | 161501ACH 161501ATB 161502ACH 161502ATB 161502ATD 161502ATR 161504ACR |
|  | fluvoxamine | 162501ATB 162502ATB |
|  | paroxetine | 209301ATB 209302ATB 209304ATR 209305ATR 209306ATR 209304ATE 09305ATE |
|  | citalopram | 428301ATB |
|  | sertraline | 227001ATB 227002ATB |
|  | Amitriptyline | 107501ATB 107502ATB 107504ATB |
|  | amoxapine | 108001ATB 108002ATB |
|  | bupropion | 428101ATB 428102ATR 428103ATR |
|  | clomipramine | 136301ACH 136302ACH |
|  | desvenlafaxine | 626401ATR 626402ATR |
|  | duloxetine | 495501ACE 495501ACH 495501ATE 495501ATB 495502ACE 495502ACH 495502ATB |
|  | imipramine | 173701ATB |
|  | milnacipran | 355801ACH 355802ACH 355803ACH |
|  | mirtazapine | 196201ATB 196201ATD 196202ATB 196202ATD 196204ATB 196204ATD |
|  | moclobemide | 196701ATB |
|  | nortriptyline | 203401ATB 203402ATB |
|  | rasagiline | 625201ATB |
|  | selegiline | 226401ATB |
|  | tandospirone | 484401ATB 484402ATB |
|  | trazodone | 242901ACH 242901ATB 242902ATB 242903ATR |
|  | venlafaxine | 247502ACR 247502ATR 247504ACR 247504ATR |
|  | vortioxetine | 628501ATB 628502ATB 628503ATB 628504ATB |

*Abbreviations*: *ICD-10*, International Classification of Diseases, 10th revision

**Table S2** ICD-10 code definitions of dementia, depression, CVD, and 14 other diseases from the Charlson comorbidity index

| **Condition** | **ICD-10 Code** | | | | | |
| --- | --- | --- | --- | --- | --- | --- |
| **Exposure Diseases** |  |  |  |  |  |  |
| Depression | F32, F33 | | | | | |
| CVD | I60, I61, I62, I63, I64, I65, I66, I67, I68, I69 | | | | | |
| **Outcome disease** |  |  |  |  |  |  |
| Dementia | F00, F01, F02, F03, G30, G31 | | | | | |
| AD | F00, G30 |  |  |  |  |  |
| VD | F01 |  |  |  |  |  |
| Non-AD or non-VD^a^ | F02, F03, G31 |  |  |  |  |  |
| **Covariates** |  |  |  |  |  |  |
| Comorbidities |  |  |  |  |  |  |
| Myocardial infarction | I21, I22, I252 | | | | | |
| Congestive heart failure | I110, I130, I132, I255, I42, I43, I50 | | | | | |
| Peripheral vascular disease | I70, I71, I731, I738, I739, I771, I790, I792, K551, K558, K559, Z958, Z959 | | | | | |
| Chronic pulmonary disease | I278, I279, J40, J41, J42, J43, J44, J45, J46, J47, J60, J61, J62, J63, J64, J65, J66, J67, J684, J701, J703 | | | | | |
| Connective tissue disorder | M05, M06, M32, M33, M34, M315, M351, M353, M360 | | | | | |
| Peptic ulcer | K25, K26, K27, K28 | | | | | |
| Mild liver disease | B18, K73, K74, K700, K701, K702, K703, K709, K713, K714, K715, K717, K760, K762, K763, K764, K768, K769, Z944 | | | | | |
| Uncomplicated diabetes | E100, E101, E106, E108, E109, E110, E111, E116, E118, E119, E120, E121, E126, E128, E129, E130, E131, E136, E138, E139, E140, E141, E146, E148, E149 | | | | | |
| Complicated diabetes^b^ | E102, E103, E104, E105, E107, E112, E113, E114, E115, E117, E122, E123, E124, E125, E127, E132, E133, E134, E135, E137, E142, E143, E144, E145, E147 | | | | | |
| Hemiplegia | G81, G82, G041, G114, G801, G802, G830, G831, G832, G833, G834, G839 | | | | | |
| Moderate or severe renal diseases | I120, I131, N032, N033, N034, N035, N036, N037, N052, N053, N054, N055, N056, N057, N18, N19, N250, Z490, Z491, Z492, Z940, Z992 | | | | | |
| Non-metastatic solid cancer^c^ | C0, C1, C30, C31, C32, C33, C34, C37, C38, C39, C40, C41, C43, C45, C46, C47, C48, C49, C50, C51, C52, C53, C54, C55, C56, C57, C58, C6, C70, C71, C72, C73, C74, C75, C76, C81, C82, C83, C84, C85, C88, C90, C91, C92, C93, C94, C95, C96, C97 | | | | | |
| Moderate or severe liver diseases | I850, I859, I864, I982, K704, K711, K721, K729, K765, K766, K767 | | | | | |
| Metastatic solid cancer | C77, C78, C79, C80 | | | | | |

*Abbreviations*: *AD*, Alzheimer's disease; *CVD*, cerebrovascular disease; *ICD-10*, International Classification of Diseases, 10th revision; *VD*, vascular dementia

^a^Includes dementia in other diseases classified elsewhere (F02), unspecified dementia (F03), and other degenerative diseases of the nervous system that have not been classified elsewhere (G31)

^b^Diabetes complicated with retinopathy, neuropathy, or renal disease

^c^Non-metastatic solid cancer including leukemia, lymphoma, and multiple myeloma

**Table S3** Observed results according to dementia subtype

| **Dementia Subtype** | **Frequency** | **Proportion** | **Cumulative Frequency** |
| --- | --- | --- | --- |
| No dementia | 229,502 | 94.74 | 229,502 |
| Alzheimer’s disease | 9,729 | 4.02 | 239,231 |
| Vascular dementia | 1,306 | 0.54 | 240,537 |
| Other dementias | 1,700 | 0.70 | 242,237 |

**Table S4** Lagged-time analysis result considering depression as a misdiagnosis or a prodrome of dementia

| **Lagged Time^a^** | **aHR^b^** | **95% CI** | | **p-value** |
| --- | --- | --- | --- | --- |
| 1 year | 1.60 | 1.51 | 1.68 | <0.0001 |
| 2 year | 1.29 | 1.21 | 1.36 | <0.0001 |

*Abbreviations:* *aHR*, adjusted hazard ratio; *CI*, confidence interval

^a^If depression occurs shortly before dementia onset (during the lagged-time period), it was categorized into the “no depression” group

^b^All models were adjusted for demographic characteristics (age, sex, residential area, and income level), cerebrovascular disease, and 14 comorbidities (myocardial infarction, congestive heart failure, peripheral vascular disease, chronic pulmonary disease, connective tissue disorder, peptic ulcer, mild liver disease, uncomplicated diabetes, complicated diabetes, hemiplegia, moderate or severe renal diseases, non-metastatic solid cancer, moderate or severe liver diseases, and metastatic solid cancer)

| **Table S5** Sensitivity analysis for the interaction effect of depression and CVD on the risk of dementia onset when depression, CVD, and dementia were defined only by the ICD-10 codes (not considering medication use) with at least 1 hospital visit | | | | | | | | |
| --- | --- | --- | --- | --- | --- | --- | --- | --- |
|  | **Risk of Dementia by Exposure, HR (95% CI)** | | | | **Additive Interaction (95% CI)** | | | **Multiplicative Interaction^a^ (95% CI)** |
|  | **No Depression**  **or CVD** | **Depression** | **CVD** | **Depression and CVD** | **AP^a^** | ***RERI*^a^** | **SI^a^** |  |
| **Model 1^b^** | 1 [Reference] | 2.14  (2.05-2.24) | 3.73  (3.62-3.84) | 5.42  (5.18-5.67) | 0.10  (0.05-0.15) | 0.55  (0.29-0.8) | 1.14  (1.08-1.21) | 0.68  (0.64-0.72) |
| **Model 2^c^** | 1 [Reference] | 2.11  (2.02-2.21) | 2.93  (2.84-3.01) | 4.44  (4.25-4.65) | 0.09  (0.04-0.14) | 0.4  (0.19-0.61) | 1.13  (1.06-1.21) | 0.72  (0.67-0.77) |
| **Model 3^d^** | 1 [Reference] | 2.06  (1.97-2.16) | 2.84  (2.75-2.92) | 4.23  (4.04-4.43) | 0.08  (0.03-0.13) | 0.33  (0.12-0.53) | 1.11  (1.04-1.19) | 0.72  (0.68-0.77) |
| **Dementia Subtype^e^** |  |  |  |  |  |  |  |  |
| AD | 1 [Reference] | 2.18  (2.04-2.32) | 2.53  (2.42-2.65) | 4.17  (3.90-4.46) | 0.11  (0.04-0.18) | 0.46  (0.16-0.76) | 1.17  (1.06-1.29) | 0.76  (0.69-0.83) |
| VD | 1 [Reference] | 2.09  (1.78-2.44) | 6.74  (6.19-7.34) | 8.37  (7.36-9.53) | 0.06  (-0.08-0.21) | 0.54  (-0.48-1.57) | 1.08  (0.94-1.24) | 0.60  (0.49-0.72) |
| Non-AD or Non-VD^f^ | 1 [Reference] | 1.94  (1.82-2.07) | 2.50  (2.39-2.62) | 3.60  (3.35-3.87) | 0.04  (-0.04-0.13) | 0.16  (-0.12-0.44) | 1.07  (0.96-1.19) | 0.74  (0.67-0.82) |
| **Age^g^** |  |  |  |  |  |  |  |  |
| 50 to 64 years | 1 [Reference] | 2.56  (2.36-2.79) | 3.67  (3.49-3.86) | 5.94  (5.47-6.44) | 0.12  (0.03-0.20) | 0.70  (0.21-1.19) | 1.16  (1.05-1.29) | 0.63  (0.56-0.71) |
| Above 64 years | 1 [Reference] | 1.88  (1.79-1.98) | 2.47  (2.38-2.57) | 3.64  (3.44-3.85) | 0.08  (0.02-0.14) | 0.29  (0.07-0.5) | 1.12  (1.03-1.22) | 0.78  (0.72-0.85) |
| **Sex^g^** |  |  |  |  |  |  |  |  |
| Male | 1 [Reference] | 2.47  (2.31-2.65) | 4.24  (4.03-4.46) | 6.08  (5.64-6.55) | 0.06  (-0.02-0.14) | 0.37  (-0.09-0.83) | 1.08  (0.98-1.18) | 0.58  (0.52-0.64) |
| Female | 1 [Reference] | 1.85  (1.74-1.96) | 2.32  (2.24-2.41) | 3.43  (3.23-3.63) | 0.08  (0.01-0.14) | 0.26  (0.04-0.48) | 1.12  (1.02-1.23) | 0.80  (0.74-0.87) |
| *Abbreviations:* *AD,* Alzheimer's disease; *AP,* attributable proportion due to interaction; *CI*, confidence interval; *CVD,* cerebrovascular disease; *HR,* hazard ratio; *RERI,* relative excess risk due to interaction; *SI,* synergy index; *VD,* vascular dementia  ^a^Null hypothesis for each interaction is AP = 0, RERI=0, SI=1, and multiplicative interaction=1  ^b^Unadjusted model  ^c^Adjusted for demographic characteristics (age, sex, residential area, and income level)  ^d^Adjusted for demographic characteristics (age, sex, residential area, and income level) and 14 comorbidities (myocardial infarction, congestive heart failure, peripheral vascular disease, chronic pulmonary disease, connective tissue disorder, peptic ulcer, mild liver disease, uncomplicated diabetes, complicated diabetes, hemiplegia, moderate or severe renal diseases, non-metastatic solid cancer, moderate or severe liver diseases, and metastatic solid cancer)  ^e^Subgroup analysis for dementia subtype was based on Model 3 (adjusted for demographic characteristics and 14 comorbidities) and conducted separately by considering the other dementia subtypes as a competing risk  ^f^Includes dementia in other diseases classified elsewhere (F02), unspecified dementia (F03), other degenerative diseases of the nervous system, and not elsewhere classified (G31)  ^g^Subgroup analyses for age and sex were based on Model 3 (adjusted for demographic characteristics and 14 comorbidities). Age or sex was not considered as a covariate in each subgroup analysis | | | | | | | | |

Fig. S1 Unadjusted survival curves for dementia onset according to the exposure diseases

A. Unadjusted survival curves for dementia onset according to the exposure of depression

**
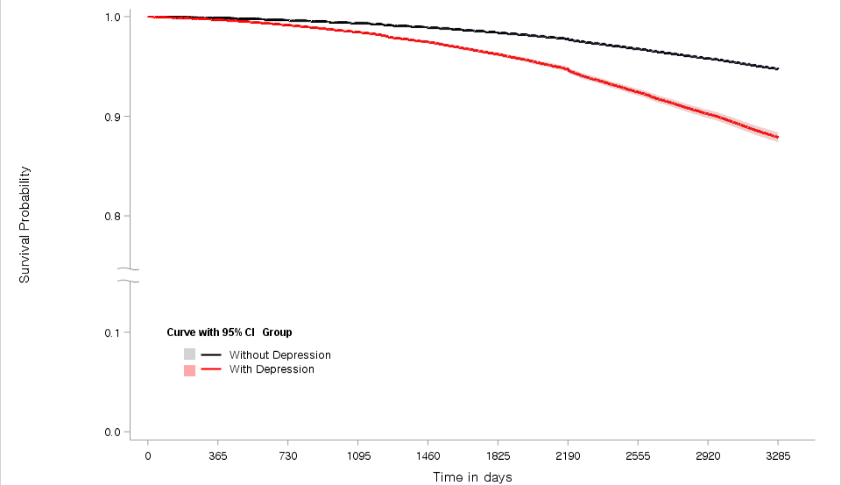
**

B. Unadjusted survival curves for dementia onset according to the exposure of CVD


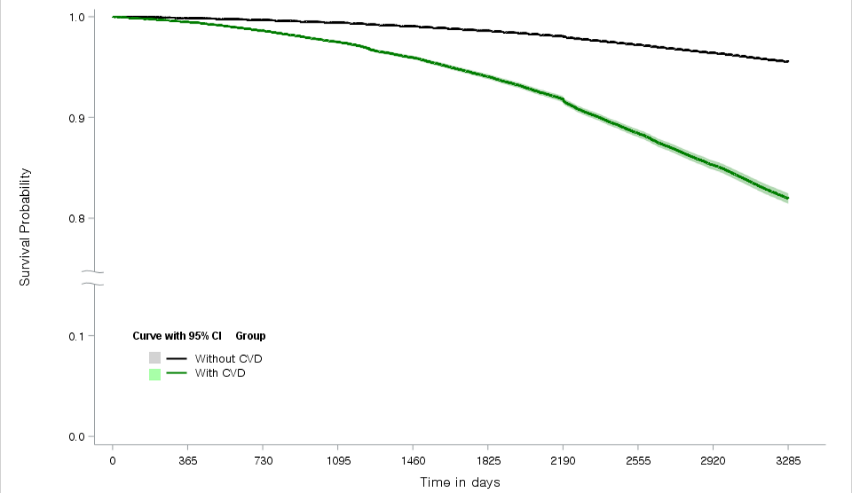


C. Unadjusted survival curves for dementia onset according to the exposures of depression and CVD


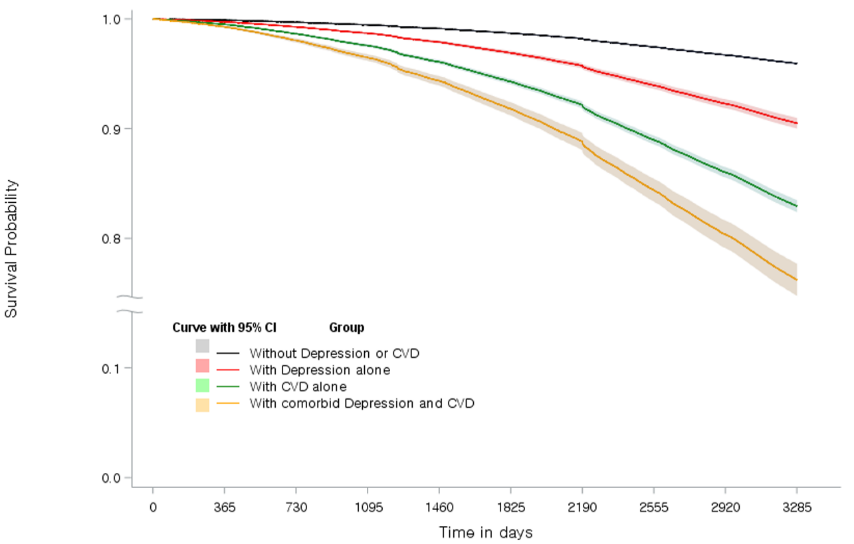

Supplement: Supplementary file 1 — Additional file 1: Table S1. Medications prescribed according to the respective dementia and depression ICD-10 codes. Table S2. ICD-10 code definitions of dementia, depression, CVD, and 14 other diseases from the Charlson comorbidity index. Table S3. Observed results according to dementia subtype. Table S4. Lagged-time analysis result considering depression as a misdiagnosis or a prodrome of dementia. Table S5. Sensitivity analysis for the interaction effect of depression and CVD on the risk of dementia onset when depression, CVD, and dementia were defined only by the ICD-10 codes (not considering medication use). Figure S1. Unadjusted survival curves for dementia onset according to exposure diseases. [file 13195_2021_800_MOESM1_ESM.docx]
